# In major joint diseases the human synovium retains its potential to form repair cartilage

**DOI:** 10.1038/s41598-023-34841-1

**Published:** 2023-06-26

**Authors:** Ernst B. Hunziker, Nahoko Shintani, Kurt Lippuner, Esther Vögelin, Marius J. B. Keel

**Affiliations:** 1 Departments of Osteoporosis and Orthopaedic Surgery, Inselspital Bern University Hospital, Freiburgstrasse 3, 3010 Bern, Switzerland; 2 Department of Osteoporosis, Inselspital Bern University Hospital, Bern, Switzerland; 3 Departments of Plastic and Hand Surgery, Inselspital Bern University Hospital, Bern, Switzerland; 4 Trauma Center Hirslanden, Clinic Hirslanden, Zurich, Switzerland; 5 Medical School, University of Zurich, Zurich, Switzerland; 6 Department of Orthopaedic Surgery, Inselspital Bern University Hospital, Bern, Switzerland

**Keywords:** Medical research, Translational research, Biologics, Regenerative medicine, Tissue engineering

## Abstract

The inner surface layer of human joints, the synovium, is a source of stem cells for the repair of articular cartilage defects. We investigated the potential of the normal human synovium to form novel cartilage and compared its chondrogenic capacity with that of two patient groups suffering from major joint diseases: young adults with femoro-acetabular impingement syndromes of the hip (FAI), and elderly individuals with osteoarthritic degeneration of the knee (OA). Synovial membrane explants of these three patient groups were induced in vitro to undergo chondrogenesis by growth factors: bone morphogenetic protein-2 (BMP-2) alone, transforming growth factor-β1 (TGF-β1) alone, or a combination of these two. Quantitative evaluations of the newly formed cartilages were performed respecting their gene activities, as well as the histochemical, immunhistochemical, morphological and histomorphometrical characteristics. Formation of adult articular-like cartilage was induced by the BMP-2/TGF-β1 combination within all three groups, and was confirmed by adequate gene-expression levels of the anabolic chondrogenic markers; the levels of the catabolic markers remained low. Our data reveal that the chondrogenic potential of the normal human synovium remains uncompromised, both in FAI and OA. The potential of synovium-based clinical repair of joint cartilage may thus not be impaired by age-related joint pathologies.

## Introduction

Pluripotent mesenchymal stem cells (MSCs) can be induced to differentiate along several distinct tissue lineages such as bone, cartilage, muscle, fat tissue etc. In the field of regenerative medicine^1^, MSCs are an attractive option for the repair of tissues that have been irreversibly damaged^2^. The human synovium—a mesenchymal tissue—harbors populations of such MSCs which are capable of undergoing chondrogenesis, osteogenesis, adipogenesis and myogenesis^3^. One of the advantages of using synovial tissues as cell source for MSC-based-tissue repair therapies is that the synovial tissue contains abundant MSC populations for chondrogenic differentiation. In general, it is believed that the differentiation potentials of MSCs decreases with aging^4^, therefore sometimes MSCs from elderly individuals might not have sufficient potential for tissue repair, in particular if bone marrow-derived MSCs^5^ are used. The general disputes on the degree of the differentiation potentials of MSCs from elderly individuals are still controversial^6^, mainly due to the use of different induction protocols for differentiation and because MSCs of different tissue sources have been dealt with; the MSC differentiation potential appears to be tissue-source dependent. However, in the case of the human, the potential of these cells for chondrogenesis seems not to decline with age^7,8^ nor of the synovial tissue itself, such as under osteoarthritic joint disease conditions^9^. In order to elucidate the chondrogenic potential of the human synovial tissues under healthy and pathological conditions, appropriate comparative studies are needed with materials derived from healthy middle-aged adults to obtain the normal base-line values and thus the basic reference data, but also from young and old patients suffering from different, but major joint diseases, such as the femoro-acetabular impingement syndrome (FAI)^10^, a disease of young adult individuals, and from patients suffering from degenerative joint disease, such as osteoarthritis (OA), like knee OA, a frequent joint pathology of mainly the elderly generations^5^. This information is of paramount importance for surgical approaches by tissue auto-transplantation of synovium for the repair of human articular defects on the basis of tissue-engineering principles, and for the identification of the normal human synovial chondrogenic potential and the limitations related to either the age of the patients and/or a major joint disease, in order to treat human patients by this principle.

It was the purpose of this study to provide the normal reference data of the human synovium for chondrogenesis of the healthy middle-aged human population, and to investigate if this capacity, as well as its degree of structural differentiation and the scope of metabolic activities are impaired or modified by major joint diseases such as FAI in young adult patients and/or OA in the elderly patient populations.

## Results

### Patients and sampling

In Table 1 an overview is presented of the total number of patients (n = 79), their gender and the numbers of samples obtained in each group (normal, FAI, OA) for histology, histomorphometry, histochemistry and immunhistochemistry, cell size measurements and gene activity measurements.

**Table 1:** Overview of patients.

Group	2 weeks	4 weeks	6 weeks
(a) Samples for histology, histomorphometry, immunhistochemistry			
FAI (n)	7	6	6
Gender	M4, F3	M4, F2	M6
Age range (years)	20–28	20–28	22–27
Mean (years)	23.3	23.0	23.7
OA (n)	7	8	7
Gender	M4, F3	M2, F6	M1, F6
Age range (years)	64–80	58–80	68–86
Mean (years)	71.1	72.4	74.4
Normal (n)	7	7	7
Gender	M6, F1	M6, F1	M6, F1
Age range (years)	21–51	21–51	21–51
Mean (years)	39.2	39.2	39.2
(b) Samples for RT-PCR			
FAI (n)	7	7	7
Gender	M4 , F3	M5, F2	M7
Age range(years)	20–29	20–29	22–29
Mean (years)	24.6	24.3	24.4
OA (n)	7	7	7
Gender	F7	M1, F6	M2, F5
Age range (years)	67–81	67–79	69–86
Mean (years)	73.7	72.7	74.4
Normal (n)	N = 7	N = 7	N = 7
Gender	M6, F1	M6, F1	M6, F1
Age range (years)	21–51	21–51	21–51
Mean (years)	39.2	39.2	39.2

♂: M, ♀: F

Total number of patients: n = 79; FAI: n = 29; OA: n = 37; Normal: n = 13.

We were able to obtain on the average 3–4 explants per patient and per experimental group for culturing and/or for morphological, histomorphological, histochemical and gene expression analyses; thus totally 12–16 explants were obtained per patient. The total time duration over which sampling was performed for the three groups was 36 months. The age spans for the normal middle-aged healthy group was 21–51 years (average 39.2 years), for the FAI group it was 20–29 years (averages in the subgroups 23–24.6 years); in the OA group the age span was 58–86 years (averages in the subgroups 71.1–74.4 years); for more details and mean age per experimental subgroup: see Table 1.

### Morphology, histochemistry, immunohistochemistry, morphometry

**Figure 1:**
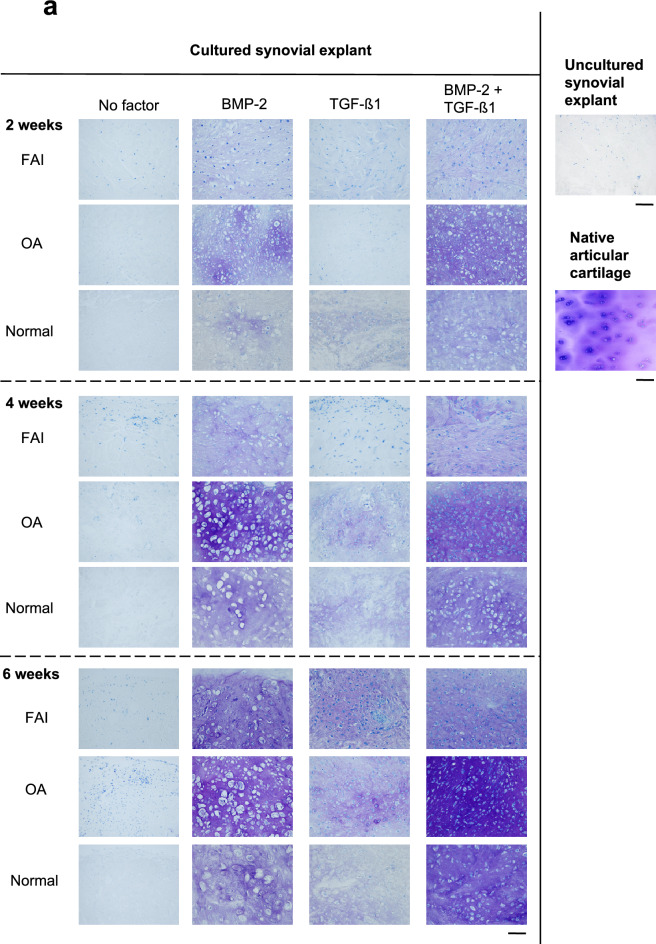

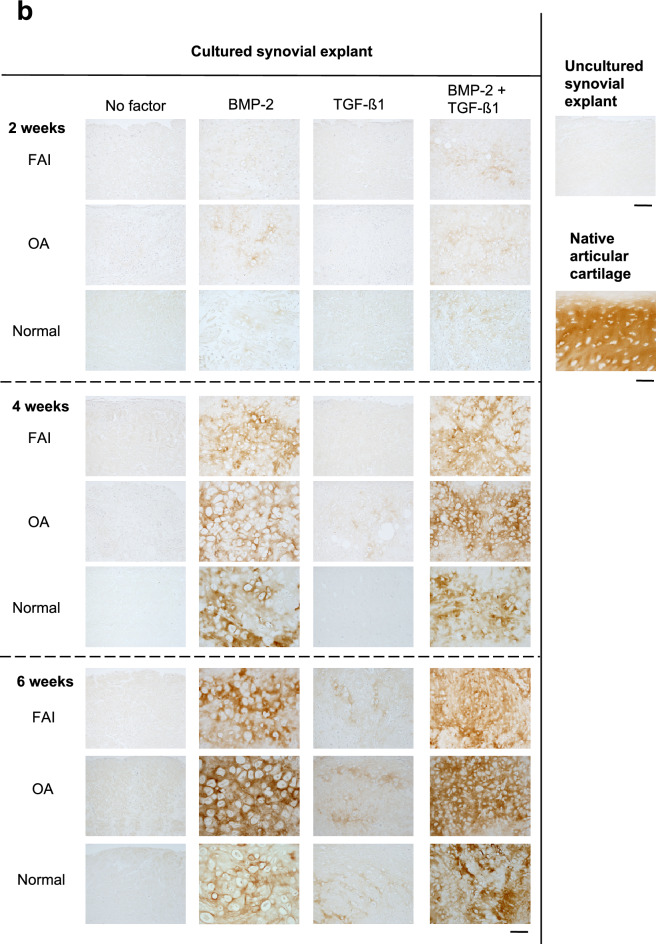
Light micrographs of sections through synovial explants. Synovial explants from FAI and OA patients as well as normal individuals were exposed for 2, 4 and 6 weeks to BMP-2 alone, to TGF-β1 alone or to a combination of BMP-2 and TGF-β1. They underwent chondrogenic differentiation, which was evidenced by the intense metachromasia of the extracellular matrix after staining with Toluidine Blue O (**a**). Explants stimulated by BMP-2 and by the combination of BMP-2 and TGF-β1 showed higher staining intensities in all groups (i.e. FAI, OA, normal). The intensities of metachromasia (matrix staining) showed a time dependent increase in the accumulation of cationic dye-positive matrix components in synovial explants stimulated with BMP-2 and/or the combination of BMP-2 and TGF-β1. Highest staining intensities were observed in the 6-week stimulation groups; the OA-group showed the most intensive staining effect. (**b**) This is a collagen-II immunostaining; it indicates too a time-dependent increase in the accumulation of collagen type-II epitope-positive materials in synovial explants stimulated with either BMP-2 and/or a combination of BMP-2 and TGF-β1. Although there are no significant differences between FAI and OA sections in all conditions, intensities of metachromasia and collagen-II immunostaining were slightly stronger in OA explants than in the FAIs or the normal individuals. Control histologies of the normal native human articular cartilage as well as a freshly-excised normal unstimulated synovial membrane are shown on the right side of the panel. Bars 100 μm.

**Figure 2:**
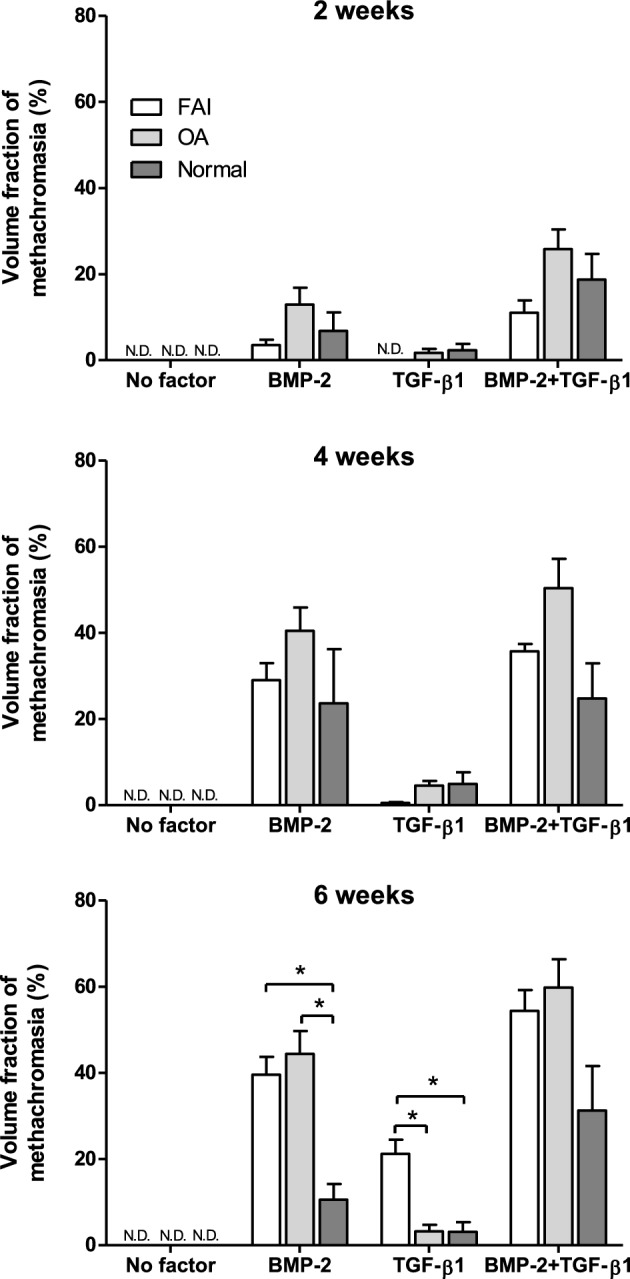
Volume fraction of metachromasia within synovial explants that had been derived from the FAI and OA patients, and the normal individuals; the percentages of tissue volumes stained by metachromasia after 2, 4 and 6 weeks culturing under defined stimulation conditions are represented. The proteoglycan productions, indicated by the intercellular matrix staining by metachromasia, were the highest in the combined stimulation groups at all time periods, and a time-dependent accumulation of positively stained matrix components was also confirmed in each growth factor condition. The OA group revealed a trend for strongest effects; but these were not significantly different at 2 and 4 weeks, but at 6 weeks for the BMP-2 groups. The TGF-β1-groups revealed the lowest volume fractions of metachromasia, except for the FAI-group at 6 weeks stimulation. Mean values are represented together with the standard error of the mean (For FAI 2 weeks: *n* = 7; 4 weeks: *n* = 6; 6 weeks: *n* = 6. For OA: 2 weeks *n* = 7; 4 weeks: *n* = 8; 6 weeks: *n* = 7. For Normal Individuals: 2 weeks: *n* = 7; 4 weeks: *n* = 7; 6 weeks: *n* = 7). *n.d*. not detected. Tukey’s multiple comparison test. *p < 0.05.

Figures 1 and 2 illustrate that in the negative control groups, i.e. the fresh uncultured synovial tissues and those cultured in the absence of growth factors, do not show any signs of chondrogenic differentiation, i.e. a complete absence of metachromasia (a blue intercellular matrix staining is absent) and the cartilage cells (chondrocytes) with their typical pericellular lacunae, are not present either; this contrasts clearly (see Fig. 1a) the presence of typical chondrocytes with their characteristic pericellular lacunae, present after treatment with BMP-2, both in the 4- and 6-weeks time point groups of the OA patients (as well as present also in all other groups (FAI and normal individuals) treated with BMP-2 or the combination of BMP-2/TGF-β1; such pericellular lacunae are indeed a characteristic feature of chondrocytes, and they are structurally revealed after chemical preservation of the tissue^11^. The two negative control groups showed identical morphological features in Fig. 1a,b.

It also is obvious in the panels of representative cases in Fig. 1a,b that the groups treated with BMP-2 alone and treated by the combination of BMP-2/TGF-β1show clear chondrogenic differentiation effects, nicely illustrated by the typical and intense blue staining results for proteoglycans in the cartilage tissue extracellular matrix (ECM), and by the presence of the rounded chondrocytes with their lacunae. The progressive increase of that staining intensity over time, i.e. from the 2 week culturing period to the 6 week time juncture is obvious in all three experimental groups (FAI, OA, normal). It is also clearly apparent that the strongest staining intensity (indicating the highest concentration of cartilage proteoglycans in the ECM) is present in the combined BMP-2/TGF-β1group. It also can be recognized that the distribution of the blue stain is somewhat inhomogenous over the section surfaces (i.e. in the different tissue areas), with the least variation, i.e. highest degree of homogeneity, present again in the groups treated by the combination of BMP-2/TGF-β1.

Figure 1a,b also illustrate that a chondrogenic differentiation of the synovial tissue into cartilage was practically absent in all three groups at the 2 weeks time juncture of culturing when stimulated by TGF-β1alone. However, after 4 and 6 weeks culturing the tissues of this group showed inhomogenous areas of beginning chondrogenic differentiation; moreover, metachromasia of the ECM remained fairly variable in intensity and homogeneity in all three groups at this early time point (2 weeks), but clear differences of appearance were attained compared to those of uncultured synovial explants and explants cultured in the absence of any growth factor. This finding contrasts the tissue differentiation effects obtained in the BMP-2 alone and the BMP-2/TGF-β1combined groups in which, after 4 and 6 weeks culturing times, ECM staining intensities were present to a degree as in the native articular cartilage control groups.

The immunoreactivity for (cartilage-specific) type II collagen (Fig. 1b) likewise reflected the findings described above for the metachromatic staining of the ECM with Toluidine Blue O. Figure 2 illustrates graphically the quantitative results of the histomorphometrical analyses of the volume fractions of metachromasia obtained in the three patient groups. The graph illustrates the increase in the volume fraction of metachromasia over time in all three patient groups with a surprising trend to be highest in the elderly (OA) group; however, this trend is statistically not significant. It is also surprising that the young (FAI) and older (OA) age groups with joint disease show tissue differentiation results that are, at 6 weeks, significantly higher than in the normal adult group in the BMP-2 treated patient pool. And in the TGF-β1pool at 6 weeks it is the FAI patients (i.e. the group of the youngest average age) that show a significantly higher tissue transformation effect; however, reverse effects (with no-significant trends) can be observed in the same patient pool at 2 and 4 weeks of stimulation/culturing.

**Figure 3:**
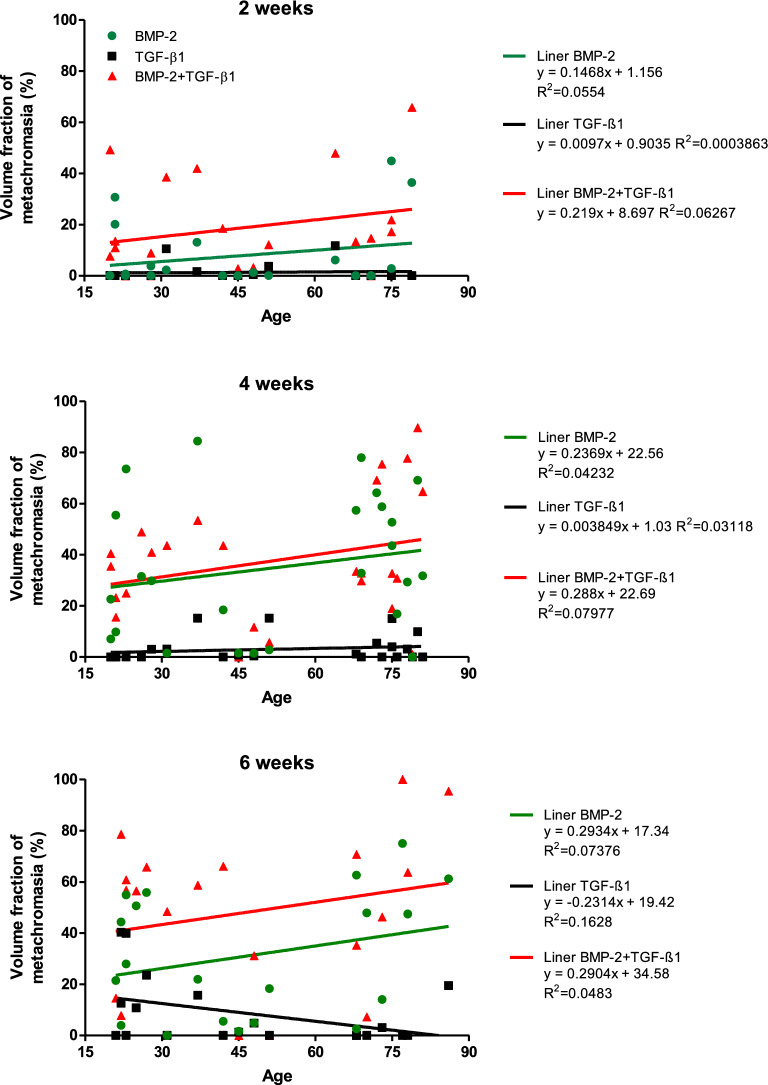
Linear regression analyses of the volume fractions of metachromasia as a function of the age of the patients and stimulation time. The data reveal that there is no significant age-related correlation, neither with the age of the patients nor with a stimulation protocol or with a period of time duration of stimulation. The data appear fairly randomly scattered.

The distribution of data of all patients (Fig. 3) illustrates in impressive random scatter of results, and confirms the independency of the metachromasia results from age and from diseases; and it also illustrates that already within the normal population this impressive scatter is physiologically present. The linear relationship computation yields R^2^ values (coefficient of determination) in very low ranges of 0.03–0.07. The lines representing the simple linear relationships illustrate a slight (non-significant) increase of transformation intensity with age (except for the TGF-β1group at 6 weeks (due to the higher degree of tissue differentiation found in the FAI group; see above). Also within each patient group (FAI, OA, normal; data not shown) no trends could be observed as a function of age (for the OA group: see^9^.

Sections of each 6 week culture group that were processed for the von Kossa staining for mineralised ECM were all negative (data not shown). No signs of terminal tissue calcification could yet be observed in any of the groups at this point in time.

### Cell size analyses

**Figure 4:**
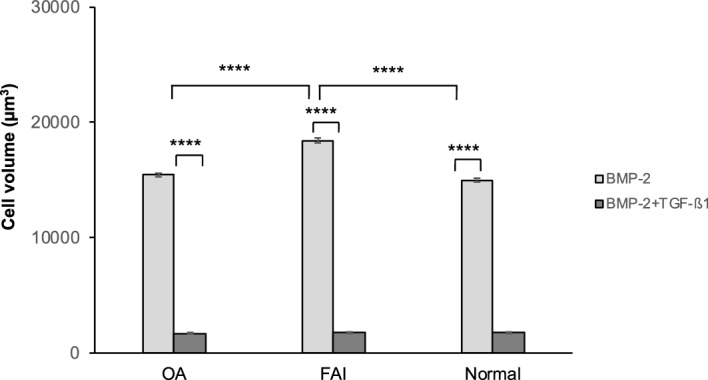
The measurements of the terminal cell volumes achieved at the maximum stimulation times reveals that in each group investigated (OA, FAI, normal individuals) that stimulation with BMP-2 results in unphysiologically high terminal cell volumes, whereas stimulation with the combination of BMP-2/TGF-β1 results in terminal cell volumes that lie in the order of magnitude of the physiological adult articular cartilage chondrocytes terminal cell volumes in the lower radial zones. ****p < 0.0001.

The mean final chondrocyte volumes attained after 6 weeks culturing period are illustrated for all three groups in Fig. 4. The mean terminal chondrocyte cell volume achieved when exposed to the BMP-2 stimulation protocol was 18,451 µm^3^ (coefficient of error (CE) = 1.14%) in the FAI group and 15,009 µm^3^ (CE = 1.11%) in the normal group of individuals, a value similar to the 15,452 µm^3^ (CE = 1.03%) in the OA group (as recently found^9^). The terminal cell volume found in the FAI group was significantly higher than that in the OA and normal groups (p < 0.0001 and p < 0.0001). And in the BMP-2/TGF-β1 stimulation protocol groups the terminal chondrocyte cell sizes achieved approximately 1736–1775 µm^3^ (CE = 3.66–3.99%) for the three groups. Between the groups subjected to the same stimulation protocol there were no significant differences encountered; thus, the two stimulation protocols (BMP-2 alone and BMP-2/TGF-β1 combined) yielded significantly different terminal cell volumes (p < 0.0001) within each group (see Fig. 4). Due to the insufficient degrees of tissue differentiation observed in the groups stimulated with TGF-β1only (absence of metachromasia, absence of chondrocytes), the final cell sizes attained were not measured (see Fig. 1a).

### Gene expression evaluation

**Figure 5:**
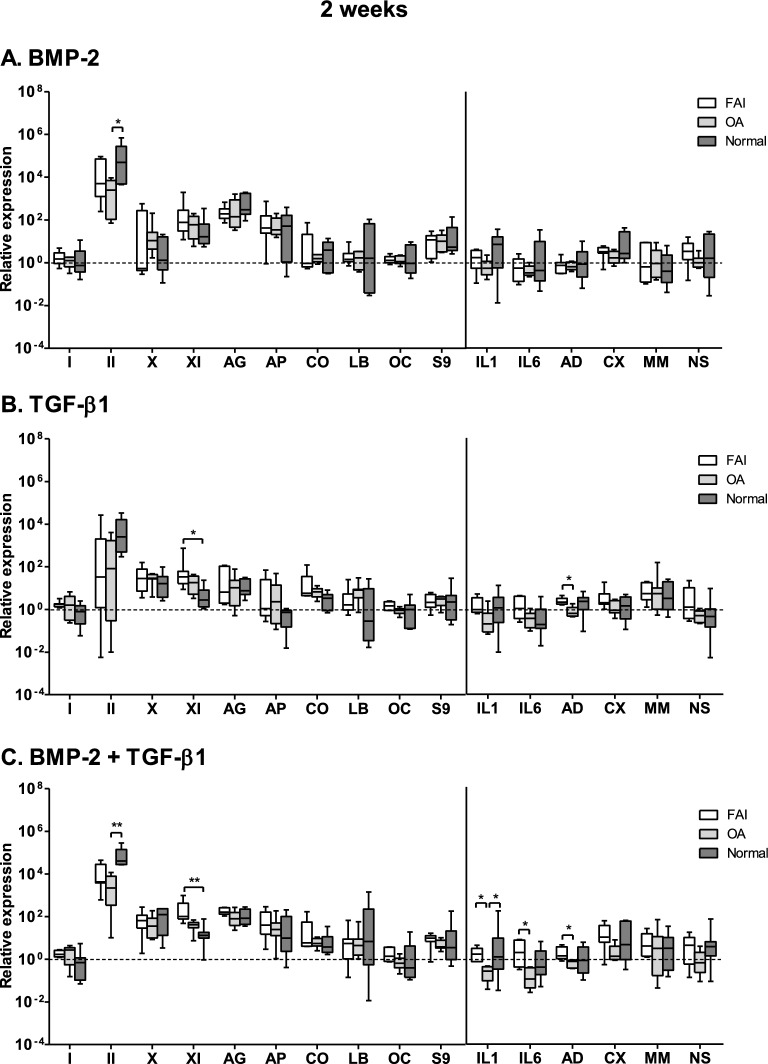
Relative changes in the gene-expression levels within the synovial explants following 2-weeks stimulation of synovial explants from FAI-patients, OA-patients and normal individuals under the defined stimulation conditions ((**A**) BMP-2; (**B**) TGF-β1; (**C**) the combination of BMP-2 and TGF-β1). Anabolic genes: *I* type-I collagen, *AG* aggrecan, *OC* osteocalcin, *II* type-II collagen, *AP* alkaline phosphatase, *S9* Sox-9, *X* type-X collagen, *CO* COMP, *XI* type-XI collagen, *LB* lubricin. Catabolic genes: *IL1* Inteleukin-1β, *CX* COX-2, *IL6* Inteleukin-6, *MM* MMP-13, *AD* ADAMTS-4, *NS i*NOS. Stimulation with growth factors induced the most profound increases in the gene-expression levels of the chondrogenic markers: collagen types II, X and XI, aggrecan, alkaline phosphatase and COMP. On the other hand, the catabolic markers were practically not stimulated compared with the anabolic markers. There were no significant differences among 2, 4 and 6 weeks culturing periods (see Figs. 6, 7). FAI group: *n* = 7; OA- group: *n* = 7; normal individuals: n = 7. *p < 0.05, **p < 0.01.

**Figure 6:**
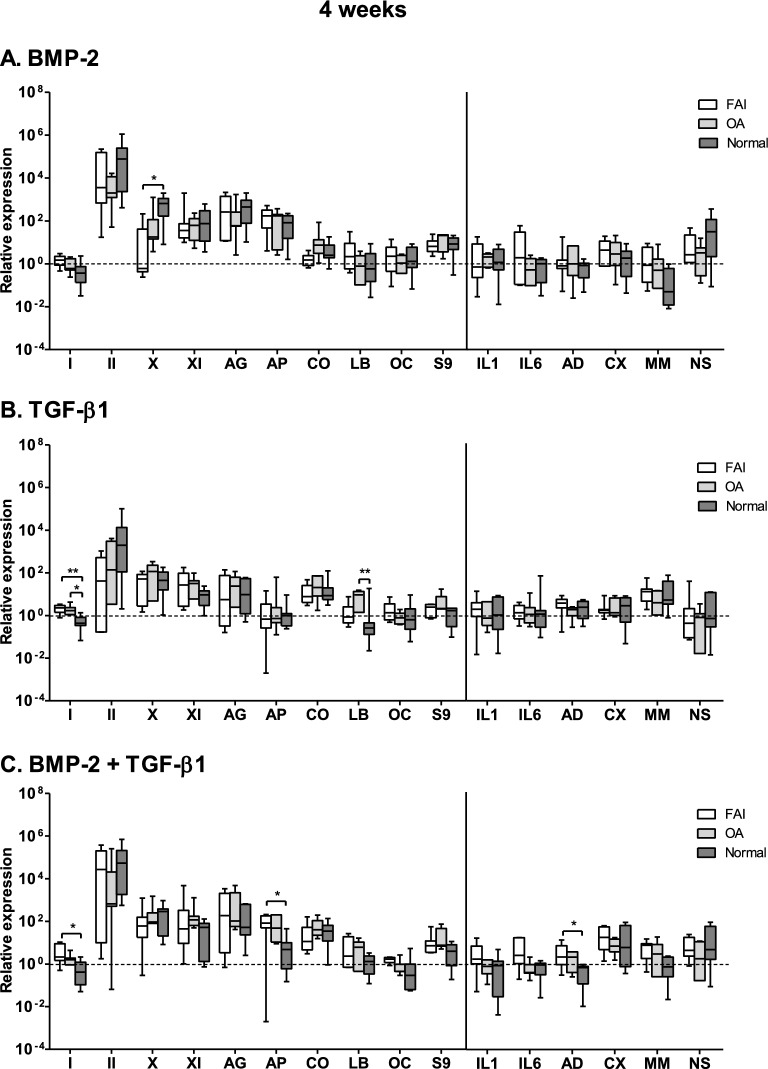
Relative changes in the gene-expression levels within the synovial explants following 4-weeks stimulation of synovial explants from FAI-patients, OA-patients and normal individuals under the defined stimulation conditions ((**A**) BMP-2; (**B**) TGF-β1; (**C**) the combination of BMP-2 and TGF-β1). Again, the gene expression levels of both the anabolic and catabolic genes follow very similar expression activity levels. Abbreviations used: see legend to Fig. 5. Mean values are represented together with the standard error of the mean. FAI group: *n* = 7; OA-group: *n* = 7; normal individuals: n = 7. *p < 0.05, **p < 0.01.

**Figure 7:**
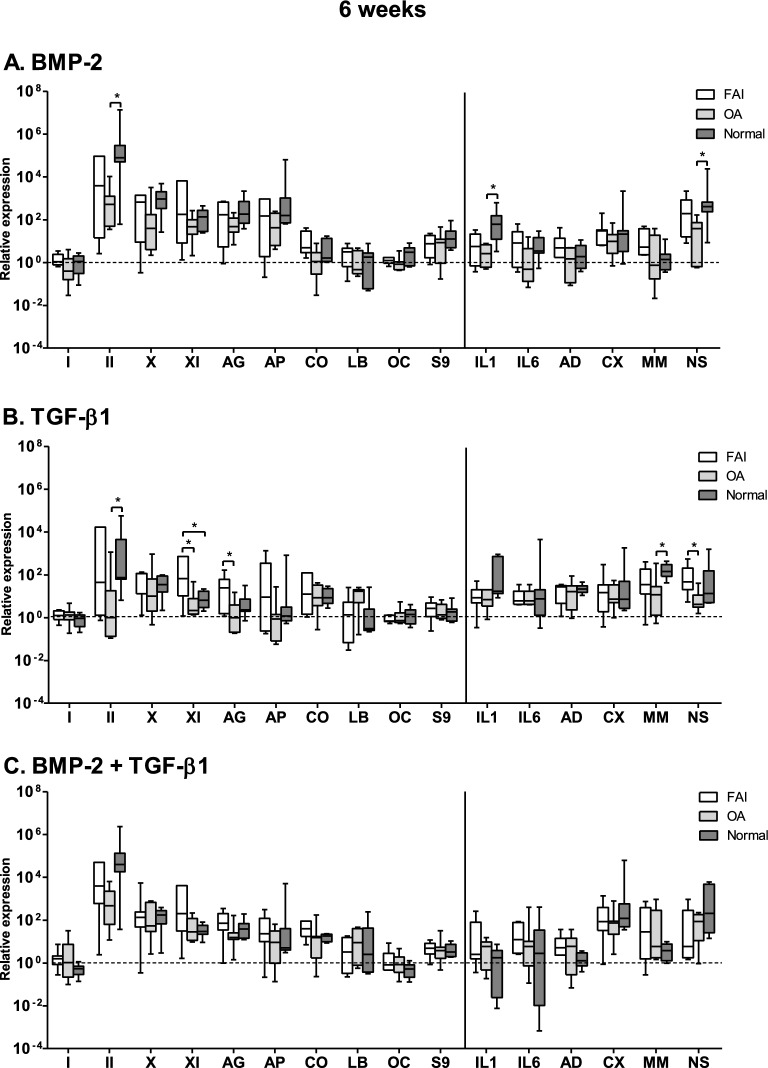
Relative changes in the gene-expression levels within the synovial explants following 6-weeks stimulation of synovial explants from FAI-patients, OA-patients and normal individuals under the defined stimulation conditions ((**A**) BMP-2; (**B**) TGF-β1; (**C**) the combination of BMP-2 and TGF-β1). Abbreviations used: see legend to Fig. 5. Mean values are represented together with the standard error of the mean. FAI group: *n* = 7; OA-group: *n* = 7; normal individuals: n = 7. *p < 0.05, **p < 0.01.

Figures 5, 6 and 7 illustrate the activities of the anabolic and catabolic gene activity results in the three patient groups as a function of culturing time and growth factor -based stimulation protocols. A view at-a-glance reveals that the gene expression levels of the collagen II gene (the marker gene for cartilage tissue) show the highest activity levels (please note that the y-axis in this plot is in a logarithmic scale) for all three time points investigated, and for all three patient groups.

It is also noteworthy that the collagen XI gene expression levels of the two groups with joint diseases (FAI, OA) show highest levels in the BMP-2/TGF-β1combined groups, at least in the initial stimulation phase at 2 weeks.

It is also quite obvious that the gene expression levels of both the anabolic and catabolic genes follow generally very similar expression activity levels for all three groups and over the whole period of time monitored, i.e. the pattern of the gene expression levels of the anabolic and catabolic genes were thus very similar in all groups and over the whole period of time investigated.

The mRNA gene expression levels of matrillin-1, IL-4 and TNF-alpha, encoding for catabolic activities, lay below the limits of detection and are not represented in Figs. 5, 6 and 7. The collagen type I gene activity levels (an unwanted gene activity in cartilage) was transitorily increased in the FAI and OA groups at the 2 week time juncture over that of the normal group, but they fell back again to the low activity levels of the normal group for these genes.

The catabolic gene activity levels remained very low at the 2 and 4 weeks time junctures; after 6 weeks stimulation they showed a slight rise in activities in all three groups following the TGF-β1only stimulation and the BMP-2/TGF-β1combined stimulation groups; however, in the TGF-β1only stimulation group all six catabolic genes showed a slight rise in activity; but in the combined stimulation groups such a slight activity raise was only the case for the cyclooxygenase-2 (CX) and the inducible nitric oxide synthase (NS) genes. However, these activity level changes are trend indications only, since statistically they were found not to reach significance levels.

A particular finding was the transient activity level increase of the IL-6 gene in the FAI group at 2 weeks in the combined BMP-2/TGF-β1stimulation group, and of the IL-1 gene activity level at 6 weeks of the normal group.

## Discussion

We previously found that synovial explants, in which the cells are maintained in their original 3D mileu^12,13^ (bovine synovium model), are able to form more abundant cartilage matrix than isolated synovial cells, even when these were cultured under 3D-conditions (e.g. in alginate^8,14^). The human synovial explants investigated in this study confirm this finding. And surprisingly, not only the synovium of the normal individuals were found here to show a high chondrogenic activity, but also synovium of joints with a major disease, both in young adults (FAI) and in elderly individuals (OA). The direct use of synovial tissue (autotransplantation) for cartilage repair purposes in a clinical setup has the great advantage to avoid the need for cell isolation and preculturing (as e.g. in approaches based on autologous chondrocyte implantation^15^, and thus could be a promising route for the repair of articular cartilage lesions, in particular also in diseased joints.

Possible reasons for the abundant formation of cartilage matrix in these explants, and in particular in the groups treated with BMP-2 alone and in the combined BMP-2/TGF-β1stimulation group, may lay in the preserved presence of the physiological pericellular matrix scaffold (ECM) that could significantly facilitate chondrogenic differentiation than other, non-joint associated and/or synthetic scaffolds (such as type I collagen, etc.^16,17^); or by the use of simply naked cells (mesenchymal stem cells (MSCs), chondrocytes, etc.) in the absence of any ECM^18^. And it had been shown before that indeed synovial tissue, under both clinicopathological and experimental conditions, is able to form spontaneously cartilage tissue in the form of tumors^19^ or as cartilage-bone-like tissues (osteophytes)^20,21^.

The trend of the synovium of the diseased joints, both in FAI and OA, to form cartilage even more readily, even though not on a significant level, than in the synovium of the normal human adults, is surprising. A speculative interpretation may be that the investigated synovia are from joint pathologies that can be associated with inflammatory episodes, which may be associated with the presence of higher numbers of cells from the monocyte/macrophage lineage, and which, in turn, is well known to be able to facilitate and/or enhance a growth factor mediated tissue differentiation potential^22^, such as those with TGF-β1 and/or BMP-2. And the presence of such local activities may indeed facilitate chondrogenic differentiation effects^23,24^.

In a recent study we found^9^ that in terminal OA (before total joint replacement) the chondrogenic potential of the synovium is maintained compared to that of the young OA patients, and thus was found to be independent of the age of the patient. However, at that time the chondrogenic differentiation potential of the synovium of the normal healthy adult persons was unknown. Is this differentiation potential reduced/impaired in both the young and the old OA patients compared to the healthy normal middle-aged population ? Or does this differentiation potential of the synovial membranes of the OA-patient persist on a high activity level, comparable to that of the healthy synovium of a normal adult individuum ? In short, the normal healthy reference level was missing. And here we provide for the first time the data for the chondrogenic potential of the synovial membrane of normal human (middle-aged) persons; and surprisingly, this potential is the same as that in young patients with FAI and is also that of elderly patients with severe OA, i.e. it is not reduced under a major joint disease condition. Given now the availability of these baseline data of the normal population we now have available a basis of comparison (and a reference data pool) that allows us to analyze other patient groups suffering from different forms of joint diseases. This is a great advantage since access to normal human data are generally a difficult task; and it was possible here only thanks to the special trauma center cases where appropriate individuals could be identified for adequate synovial tissue availability.

From the point of view of clinical practice it is encouraging to see that beside the absence of a decline of the chondrogenic potential of the isolated human synovial MSCs with age^7,8^ there is also an absence of such a decline for the synovium under major joint disease conditions, as found in this study.

For example, in FAI it was found that isolated MSCs from human synovium maintain a high proliferation potential^25,26^which may vary as a function of local topological origin (niche effect)^25^. However in these studies it was not known if the activity level of this differentiation potential relates to a level of differentiation as it is present in a normal human synovial joint, or not; the appropriate control group was obviously not available. Moreover, the synovial tissue of FAI joints tends to form spontaneously cartilage-like tumors^27–29^, which may also have been a confounding factor.

However, it cannot be excluded that some joint diseases are indeed associated with such a decline, in particular when the disease affects primarily the synovium itself, such as in rheumatoid arthritis^30^ or in special forms of FAI^31,32^ that differ from the more frequent degenerative forms of FAI^24^, or also in OA. The diversity of the local pathology in FAI in the labro-acetabular complex illustrates the high degree of locally-contained niche-type of physiological processes^33^. This may be one of the reasons why surgical treatment of this disease is not a straight-forward simple approach^34^, but requires sophisticated evaluations and case-specific measures.

It is conceivable that the great potential of the synovium to undergo chondrogenesis in a FAI affected joint that is suffering simultaneously from significant inflammatory episodes^32^, compared to the immunogenically-induced inflamed synovium, as present in various chronic autoimmune diseases and/or rheumatoid arthritis, the situation respecting its chondrogenic potential may be different. This remains to be clarified. Nevertheless, on the basis of the great potential of synovium-derived mesenchymal stem cells (MSCs) to induce the formation of novel tissues in regenerative medicine, in particular in orthopaedics and in rheumatology^35^, respecting patients with typically localized or circumscribed articular cartilage defects (such as they occur after trauma, in focal forms of OA, in osteochondritis dissecans, etc.), and such patients in particular could potentially greatly benefit from an autologous synovial MSC-based cartilage repair approach.

OA often is associated with the formation of osteophytes (OSP) that form preferentially in the joint periphery. They often are associated with the periosteum and/or the synovial membrane. Generally the OSPs are localized in the synovial tissue space^36,37^: and it is the MSCs of these tissues that form the cartilage and bone tissue of the osteophytes^38^. Experimental data point out that the main cell source for their formations lie indeed in the synovium^39,40^, and are associated with the local internal release of TGF-β1 and/or BMP-2^41–43^ during the process of the active disease. And even though the physiological role of the OSPs remains unclear (desirable role: stabilizing the joint biomechanically? Undesirable role: limiting the range of motion^21,44^?), it is well documented that they are formed initially by cartilage tissue formation^41,42^, a cartilage that undergoes a continuos growth, initiating subsequently enchondral ossification processes and the formation of bone tissue^39,41,42^. The positive aspects of these data are that the synovium of OA joints maintains also physiologically a strong chondrogenic potential during the disease process, even though it may be of an inflammatory nature. However, the use of this synovium with OSP-activity is associated with the danger that the newly formed chondrocytes continue their physiological downstream differentiation process into terminal hypertrophy, cartilage mineralization and enchondral bone tissue formation, which would be very undesirable activities when using the synovium of OA joints for tissue engineering purposes where a stable articular cartilage tissue must be generated, and the downstream differentiation process needs to be arrested in a pre-terminal phase. As it was pointed out in the “Methods” section this is achieved by the addition of TGF-β1 at low activities^13^; an alternative cell-based way would be the addition of mesenchymal stem cells (MSCs), based on paracrin and co-culturing mechanisms^45^.

During the ontogenic maturation process the articular cartilage tissue fulfills physiologically a dual function: it acts on one side as articular cartilage proper and provides the practically frictionless movement in the joints between the two adjacent bones and it transfers load from one skeletal element to the another one, but it also acts, on the other hand, during postnatal growth as a superficial growth plate, regulating the bone growth of the epiphysis of long bones, i.e. fulfills a dual functionality at the same time^14^. When reaching adulthood, the growth plate-like activity of the articular cartilage is arrested, and the columnar anisotropic tissue organization is maintained. The articular cartilage radial zone corresponds to the maturation zone and early hypertrophic zone of the growth plate^46,47^, and chondrocytes of these zones cease further differentiation processes into terminal hypertrophy at this point in time of skeletal development. And in this way the extensive terminal hypertrophy process, needed for epiphyseal growth, is arrested as well as are tissue mineralization processes and activities of new bone formation. The radial zone cells of the adult human articular cartilage^48^ are characterized by a specific cell size and volume, and this is significantly smaller than the chondrocyte cell volumes attained during terminal hypertrophy. Given this background measurements of cell size and the histochemical staining for calcification were included in this study. And indeed, it was only possible to arrest the chondrocyte differentiation process at the appropriate cell size stage of adult human articular cartilage^49^ by the use of the combined stimulation protocol with BMP-2/TGF-beta 1. The effective use of this combined growth factor pair for this purpose had been shown earlier with bovine materials^13^, and it seems to work also for the human tissue, as documented here. And an alternative approach to achieve this goal would be the use of mesenchymal stem cells (MSCs) with their well-documented paracrine activities^50^ (and operating on a co-culturing type of mechanism), and their potential to prevent terminal chondrocyte hypertrophy^45^.

These data illustrate also the usefulness of the bovine in vitro model and studies^13,14^ that are able to help to reduce the number of animal experiments in the interest of the 3R philosophy^51^.The use of only BMP-2 lead to the formation of hypertrophic chondrocytes in their large terminal size, and eventually matrix mineralization may occur (not yet observed after 6 weeks culturing). Tissue organization of such huge terminal chondrocytes is, on the other hand, also associated with biomechanical tissue properties that are inadequate and insufficient to fulfill the demands of the adult human articular cartilage (much too low volume density of the cartilage matrix, being mainly responsible for the mechanical tissue properties^52^); these implications are another important reason to achieve a controlled arrest of the downstream differentiation of the newly generated cartilage tissue.

The gene expression activity levels demonstrate the general positive correlation of anabolic cartilage gene activities with the formation of the new cartilage tissue. And the catabolic gene activities stayed quite low in all three groups. Obviously these gene activity profiles and patterns do not allow to differentiate between different cartilage types and/or degrees of differentiation. A useful example to illustrate this limitation is the activities of the type X collagen gene, associated generally with hypertrophy and terminal differentiation. Even though the three experimental groups were found in this study to be morphologically and histomorphometrically significantly different from one another, the collagen X gene expression levels remained practically on the same relatively low activity level in all three stimulation groups over the whole time period (6 weeks) investigated (no significant differences between them). The morphological, morphometrical and histochemical analyses are thus indispensable to provide the desired information of degree of tissue differentiation and type.

Alkaline phosphatase is likewise a marker of terminal chondrocyte hypertrophy (and matrix mineralization)^53^. And the activities of this gene remained also on very low levels in all three experimental groups, i.e. not reflecting the tremendous differences in degrees of hypertrophy attained under the three different stimulation protocols. On the other hand, this finding can also be interpreted positively in the sense that further downstream differentiation of the newly generated chondrocytes most likely will not happen so that the unwanted stage of tissue mineralization may never be reached. However, without additional long-term investigations this remains uncertain.

An analysis of the posttranslational expression of gene products would be another useful attribute to possibly provide additional information on the gene products and their available quantities. However, also these, would not be able to provide the very essential information on the structural tissue organization, on the histophysiology and the ultimate result respecting required biomechanical tissue properties. Even though such additional experiments are desirable to complete our picture of the attainable tissue quality, they may be undertaken only now in a more targeted way on the basis of the structural and functional tissue organization data provided in this study.

The really encouraging gene activity results obtained here are those for type II collagen that seemed to be the dominating gene activities in the three patient groups. Also the fact that type I collagen gene activities remained low throughout the investigated time period is quite an encouraging finding from the point of view to ultimately employ this newly generated tissue for clinically applied tissue engineering purposes.

The high gene expression activity levels found for aggrecan, a typical and essential structural and functional component of the cartilage tissue matrix, nicely point in the same direction as the collagen II gene activities. However, these activity levels are not as high as expected as for the type II collagen gene. And again, the fact that the TGF-β1stimulated groups show gene activity levels for aggrecan similar to those of the other two groups, even though the structural and morphometrical data are fundamentally different, illustrates the limited usefulness of the gene activity analysis if taken on an isolated basis without the different additional structural and quantitative investigations and characterizations. Clearly, the TGF-β1stimulated groups showed practically no differentiation effects into cartilage, but the aggrecan gene activity levels failed to indicate that.

An encouraging finding was indeed that the catabolic gene activities all remained at low levels throughout the experimental time investigated. This finding can be interpreted in a way that the usual tissue remodeling activities start early on and consistently, even in initial phases, in novel tissue formation stages and under growth. The low catabolic gene activity levels may indicate that the newly formed tissue is a stable product, undergoing physiological remodeling only, and is not driven towards a dominating degradation and tissue destruction pathway.

In conclusion, our data reveal that in young and elderly patients alike, suffering from different major joint diseases, their synovium can be equally well induced to undergo chondrogenesis as in normal individuals. These findings are encouraging to further develop this concept for clinical applications for articular cartilage repair in patients suffering from various joint diseases.

## Materials and methods

### Tissue preparation, growth factors and explant culturing

Synovial tissue was obtained (surgical waste material) from three patient groups: the first group consisted of normal adult individuals who had suffered from severe motorbike accidents with fractured hips and traumatized/dislocated hip joints that needed reconstructive surgery and re-strengthening of the joint capsules and synovial membranes, whereby synovial tissue became available as surgical waste material (n = 13 patients; the age span was 21–51 years; mean 39 years). The second group consisted of young adult individuals who suffered from severe FAI syndromes that required femoro-acetabular hip impingement surgery. The age span in this group was between 20 and 29 years (n = 29 patients; mean age = 23.9 years). The third group consisted of patients that were undergoing total hip replacement due to severe primary (terminal stage) osteoarthritis (the synovial tissue again became available as surgical waste material, as was the case also in the FAI group). The patient`s age span was from 58 to 86 years; n = 37 patients; mean age: 73.2 years). This group had been part of a recent study the authors performed in which the possible role of age in OA patients plays a role respecting the synovial chondrogenesis potential (young OA patients versus old OA patients; c.f. Hunziker et al.^9^). Normal articular cartilage as a reference tissue could be obtained only from few selected cases of the trauma group where it became available as surgical waste material (since in most cases normal joint cartilage could be re-used for reconstruction purposes). Normal articular cartilage was identified as areas in the joints with an intact articular cartilage surface structure of a structural continuity without the presence of any fissures, fibrillations, focal erosions and local swellings with uneven height, i.e. without any of the typical structural changes occurring in early osteoarthritis^54^. From the OA and the FAI groups, only few locations of the removed joint tissues that showed a normal articular cartilage appearance were available for investigation. In all three patient groups the study exclusion criteria applied were chemotherapy, any systemic disease (such as diabetes, autoimmune diseases, etc.), septic arthritis, intraarticular use of steroids, viscosupplementation etc.), or long-term pain therapy.

In summary, a total number of 79 patients was available for this study. After synovial tissue removal, the explants were cultured under the following conditions (for 2, 4 and 6 weeks): no growth factor (control), 2000 ng/ml BMP-2, 10 ng/ml TGF-β1 and the combination of 2000 ng/ml BMP-2 and 10 ng/ml TGF-β1 (in order to prevent terminal differentiation to chondrocyte hypertrophy and matrix mineralization^13^; negative control groups, i.e. fresh uncultured tissue and tissues cultured in the absence of growth factors (for each time period investigated), were also established. The differentiated tissue of each experimental group was then analyzed for its cartilage properties morphologically (chondrocyte/lacunae formation) and on histochemical (volume fraction of metachromasia), as well as on immunohistochemical (Type-II collagen) grounds; the final cell volumes achieved after 6 weeks culturing period were also measured; in addition, the expression profiles of anabolic and catabolic marker genes were investigated^9^. In the gene expression analysis (see below) the control group is not shown separately in the “Results” section since it was used for the ratio computation of the gene activity levels (for more technical details: see Supplementary Information).

### Ethical approval and consent to participate

Informed consent was obtained from all the individual patients to donate their surgical waste material for the present study. An approval by the ethical commission of the medical faculty (medical school) of the University of Bern was also obtained. All tissue retrieval procedures were carried out strictly in accordance with the relevant guidelines and regulations.

### Histomorphometry, histochemistry and immunohistochemistry

At the end of each culturing period (2, 4 or 6 weeks), a portion of the specimens was processed for the histomorphometric quantification of metachromasia (sulfated proteoglycans) and the estimation of the terminal cell size (6 weeks culturing subgroup), after staining with Toluidine Blue O, and for the immunhistochemical demonstration of type-II collagen (negative controls: fresh uncultured synovium and cultured synovium (in the absence of growth factors); for technical details see Supplementary Information, and Shintani et al.^12,13^).

From each 6 week culturing group randomly selected sections were chosen for von Kossa staining in order to demonstrate possible tissue mineralization activities that may occur during preterminal/terminal chondrocyte differentiation processes. Staining was performed according a standard protocol^55^. Positive controls (cultured calcified arterial plaque material; kindly provided by the Institute of Pathology, University of Bern) were established to assure the adequacy of the staining method chosen.

### Isolation of RNA, reverse transcription and real-time PCR analysis

At the end of each culturing period (2, 4 or 6 weeks), the portion of the samples that were not used for histomorphometrical and immunohistochemical evaluations were subjected to an RT-PCR analysis to determine the gene-expression levels of key anabolic cartilage markers [collagen types I, II, X and XI, aggrecan, alkaline phosphatase, cartilage oligomeric matrix protein (COMP), lubricin, matrilin-1, osteocalcin and Sox-9] and of a panel of catabolic factors [interleukins (IL)-1β, -4 and -6, a disintegrin and metalloproteinase with thrombospondin motifs 4 (ADAMTS-4), cyclooxygenase-2 (Cox-2), inducible nitric oxide synthase (*i*NOS), matrix metallopeptidase-13 (MMP-13) and tumor necrosis factor alpha (TNF-α)]; for details (and abbreviations): see legends to Figs. 5, 6 and 7, as well as the Supplementary Information.

### Statistical analyses

All statistical analyses were conducted using Prism 8 (version 8.4.3; GraphPad Software, San Diego, CA, USA). For details see Supplementary Information.

## Supplementary Information

Supplementary Information.

## Data Availability

All data and supportive data of this study are available on an unrestrictive basis. For such requests please contact Ernst B. Hunziker, MD, PhD, at the following email address: ernst.hunziker@dbmr.unibe.ch.
