# Supplementary material for: In major joint diseases the human synovium retains its potential to form repair cartilage
Source: Sci Rep. 2023 Jun 26;13:10375. doi: 10.1038/s41598-023-34841-1 (PMC10293277; doi:10.1038/s41598-023-34841-1)
Supplement: Supplementary file 1 — Supplementary Information. [file 41598_2023_34841_MOESM1_ESM.pdf]

Supplementary Information

Details of Materials and Methods

Tissue preparation and general culturing

Quadratic pieces of tissue with a side length of approximately 2 mm were prepared and sandwiched between two layers of agarose gel [upper layer: 0.5% (0.75 ml); lower layer : 1.0% (0.25 ml)], which was diluted with Dulbecco's Modified Eagle Medium [DMEM (Sigma-Aldrich, St. Louis, MO, USA)], as previously described [1, 2]. The explants (maintained in 24-well plastic plates) were bathed with 1 ml of high-glucose DMEM (Invitrogen, Carlsbad, CA, USA) containing 1% ITS-Premix (BD Biosciences, San Diego, CA, USA), 0.35 mM proline (Sigma-Aldrich), gentamicin (50 mg/ml) (Invitrogen) and ascorbic acid 2-phosphate (25 mg/ml) (Sigma-Aldrich). They were incubated at 37 °C in an atmosphere containing 5% CO2. Culture media were changed every two days.

Specific culturing conditions

The small pieces of tissue specimens were prepared from the human synovial tissues of each donor type, i.e. the normal(trauma cases), the FAI and the OA patients; donor-intrinsic controls of normal articular cartilage for intrinsic normalization purposes were obtained from structurally normal articular cartilage (AC) areas in the removed joints of the OA cases, and from the normal small AC fragments (that could not be re-used) in the trauma cases, and from the removed AC fragments from the joint periphery of the FAI patients undergoing the reconstructive FAI -surgical protocol for the hip [3].

All specimens were cultured under the following conditions (for 2, 4 and 6 weeks): no growth factor (control), 2000 ng/ml BMP-2, 10 ng/ml TGF-β1 and the combination of 2000 ng/ml BMP-2 and 10 ng/ml TGF-β1. After the cultivation of specimens, some of them were used for the histomorphometrical analyses with Toluidine Blue O (quantitative histochemistry and cell size measurements), for immunohistochemistry with collagen type-II antibody, for histochemical assessment by the von Kossa staining [4], and others were used for gene expression analysis.

Quantitative histochemistry and immunohistochemistry

The synovial explants were chemically fixed with a 2% solution (v/v) of paraformaldehyde in phosphate buffered saline [PBS (pH 7.4)] for 2 hours at ambient temperature, rinsed three times with PBS, dehydrated in ethanol, and embedded in paraffin. 5-µm-thick sections were prepared from the embedded material [1]. For the histomorphometric quantification of metachromasia, the sections were stained with 1% Toluidine Blue O (pH 2.5) for 5 minutes and then mounted in Entellan® (Merck, Darmstadt, Germany) prior to photography at a final magnification of 40x in a Nikon Eclipse E1000 light microscope (Nikon, Tokyo, Japan). The volume fraction of metachromasia was determined from the light micrographs by the point-counting technique, which was applied in accordance with stereological principles according to Gundersen et al [5].

The von Kossa staining was performed according to a standard protocol [4], yielding mineralized materials in black colour.

For the immunhistochemical demonstration of type-II collagen, the sections were first exposed to hyaluronidase [H-6254; Sigma-Aldrich (1 mg/ml of sodium acetate buffer, pH 5.5)] for 30 minutes at 37 °C to activate the antigen. After rinsing with PBS for 5 minutes, they were incubated first with blocking buffer (1.5% equine serum in 3% skimmed milk) for 30 minutes at ambient temperature, and then with the avidin-biotin complex for 15 minutes, likewise at ambient temperature. Thereafter the sections were exposed to the primary antibody against type-II collagen (clone CII C1; Hybridoma Bank, Iowa City, IA, USA (diluted 1:25,000 in PBS containing 3% skimmed milk) for 1 hour at 37°C in a humid chamber. After rinsing, the sections were exposed to the secondary antibody (Vecstatin ABC Kit, Vector Laboratories, Burlingame, CA, USA) for 30 minutes at ambient temperature. Endogenous peroxidase activity was blocked with hydrogen peroxide. Immunoreactivity was enhanced by applying first the avidin-biotin-peroxidase complex (Vector Laboratories) and then biotinyl tyramide (Perkin Elmer, Waltham, MA, USA). To visualize immunoreactivity, the sections were reacted with diaminobenzidine (DAB Kit, Vector Laboratories, Newark, Cal., USA). Cell nuclei were counterstained with haematoxylin. The sections were evaluated and photographed in a Nikon Eclipse E1000 light microscope [2, 6]. Due to the usual intrinsic variation of immunhistochemical results these data were analysed semi-quantitatively using intrinsic normal control signals and assessed subjectively, relative to the signal obtained from normal articular cartilage of the same donor.

Terminal Cell Size Measurements

The mean volumes of the cartilage cells at the end of the 6-week culturing period were estimated using the point sampled intercept method [7], and systematic random sampling strategies [8]were applied. A total of 1800 – 2000 cell profiles were measured per experimental group.

Isolation of RNA, reverse transcription and the real-time PCR analysis.

The total amount of RNA was isolated using the RNeasy Micro Kit (Qiagen, Basel, Switzerland) in accordance with the manufacture's standard protocol, which included a deoxyribonuclease-catalyzed digestion step. The concentrations of mRNA were measured in a Nano Drop spectrophotometer ND-1000 (NanoDrop Technologies, Wilmington, DE, USA). The samples of mRNA were stored at -70 °C and then subjected to reverse transcription (200-ng aliquots) using an ImProm-II- Reverse Transcription (Promega, Madison, WI, USA), in accordance with the manufacturer's instructions. Samples of cDNA were diluted 1:10 in nuclease-free water and stored at -20° C. 1-ng aliquots of cDNA were employed for the relative quantification of RNA by an RT-PCR analysis using an ABI PRISM 7900 Sequence Detection System (Applied Biosystems, Foster City, CA, USA) and 96-well PCR plates (Thermo Fisher Scientific, Rockford, IL, USA). The sequences of the primers and probes and their concentrations are given in Tables 1 and 2. The probes were labelled with 6-carboxy-fluorescein at the 5' end and with Eclipse® Dark Quencher at the 3' end. Primer and Probes were used at the given final concentrations (Tables 1 and 2) in a volume of 25 µl. In preparation for the RT-PCR analysis, 25-µl aliquots of the primers, probes, cDNA and qPCR MasterMix Plus (Eurogentec, Seraing, Belgium) were subjected to an initial 10-minute denaturation step at 95 °C, followed by 45 cycles of a 15-second denaturation at 95 °C, and a 60-second extension at 60 °C. The reactivity of the primers and probes was confirmed using samples of cDNA that had been prepared from human cartilage or the stimulated human synovial explants. The reactivity of the primer and probe for IL-4 was confirmed using cDNA that had been prepared from human blood. The levels of mRNA were quantified relative to those for 18S rRNA using the comparative cycle-threshold method [1]. For these calculations, the gene-expression levels in unstimulated synovial explants served as the basis for comparison [9].

Statistical Analyses

For volume fractions of metachromasia and terminal cell volumes, comparisons between two sets of data were statistically evaluated by unpaired *t*-tests. Comparisons between multiple groups were evaluated by one-way ANOVA and then by implementing Tukey's multiple comparison test. For gene expressions, comparisons between multiple groups were evaluated by Kruskal-Wallis test and then by implementing Dunn's post hoc test. The correlation between the age of patients and the volume fraction of metachromasia was determined using R square values calculated by a linear regression.

References for Supplementary Information

1. Kurth, T., et al., *Chondrogenic potential of human synovial mesenchymal stem cells in alginate*. Osteoarthritis Cartilage, 2007. **15**(10): p. 1178-89.
2. Shintani, N. and E.B. Hunziker, *Differential effects of dexamethasone on the chondrogenesis of mesenchymal stromal cells: influence of microenvironment, tissue origin and growth factor*. Eur Cell Mater, 2011. **22**: p. 302-19; discussion 319-320.
3. Goto, T., et al., *The state of the art in arthroscopic hip surgery*. J Med Invest, 2014. **61**(3-4): p. 226-232.
4. Schneider, M.R., *Von Kossa and his staining technique*. Histochem Cell Biol, 2021. **156**(6): p. 523-526.
5. Gundersen, H.J., et al., *Some new, simple and efficient stereological methods and their use in pathological research and diagnosis*. APMIS, 1988. **96**(5): p. 379-394.
6. Shintani, N., K.A. Siebenrock, and E.B. Hunziker, *TGF-beta1 enhances the BMP-2-induced chondrogenesis of bovine synovial explants and arrests downstream differentiation at an early stage of hypertrophy*. PLoS One, 2013. **8**(1): p. e53086 (https://journals.plos.org/plosone/article?id=10.1371/journal.pone.0053086).
7. Gundersen, H.J. and E.B. Jensen, *Stereological estimation of the volume-weighted mean volume of arbitrary particles observed on random sections*. J Microsc, 1985. **138**(Pt 2): p. 127-142.
8. Gundersen, H.J. and E.B. Jensen, *The efficiency of systematic sampling in stereology and its prediction*. J Microsc, 1987. **147**(Pt 3): p. 229-263.
9. Livak, K.J. and T.D. Schmittgen, *Analysis of relative gene expression data using real-time quantitative PCR and the 2(-Delta Delta C(T)) Method*. Methods, 2001. **25**(4): p. 402-408.
